# Direct and Indirect Effects of Climate on Demography and Early Growth of *Pinus sylvestris* at the Rear Edge: Changing Roles of Biotic and Abiotic Factors

**DOI:** 10.1371/journal.pone.0059824

**Published:** 2013-03-26

**Authors:** Raquel Benavides, Sonia G. Rabasa, Elena Granda, Adrián Escudero, José A. Hódar, Jordi Martínez-Vilalta, Ana M. Rincón, Regino Zamora, Fernando Valladares

**Affiliations:** 1 Department Biogeografía y Cambio Global, Museo Nacional de Ciencias Naturales-CSIC, Madrid, Spain; 2 Área de Biodiversidad y Conservación, Universidad Rey Juan Carlos, Móstoles, Madrid, Spain; 3 Departamento Ecología, Universidad de Granada, Granada, Spain; 4 CREAF, Cerdanyola del Vallès, Barcelona, Spain; 5 Universitat Autònoma Barcelona, Cerdanyola del Vallès, Barcelona, Spain; 6 Departamento Protección Vegetal, Instituto de Ciencias Agrarias-CSIC, Madrid, Spain; University of Tartu, Estonia

## Abstract

Global change triggers shifts in forest composition, with warming and aridification being particularly threatening for the populations located at the rear edge of the species distributions. This is the case of Scots pine (*Pinus sylvestris*) in the Mediterranean Basin where uncertainties in relation to its dynamics under these changing scenarios are still high. We analysed the relative effect of climate on the recruitment patterns of Scots pine and its interactions with local biotic and abiotic variables at different spatial scales. Number of seedlings and saplings was surveyed, and their annual shoot growth measured in 96 plots located across altitudinal gradients in three different regions in the Iberian Peninsula. We found a significant influence of climate on demography and performance of recruits, with a non-linear effect of temperature on the presence of juveniles, and a positive effect of precipitation on their survival. Abundance of juveniles of *P. sylvestris* that underwent their first summer drought was skewed towards higher altitudes than the altitudinal mean range of the conspecific adults and the optimum elevation for seedlings' emergence. At local level, light availability did not influence juveniles' density, but it enhanced their growth. Biotic interactions were found between juveniles and the herb cover (competition) and between the number of newly emerged seedlings and shrubs (facilitation). Results also highlighted the indirect effect that climate exerts over the local factors, modulating the interactions with the pre-existing vegetation that were more evident at more stressful sites. This multiscale approach improves our understanding of the dynamics of these marginal populations and some management criteria can be inferred to boost their conservation under the current global warming.

## Introduction

Climate is the primary filter that determines where a specific species can thrive [1], [2]. Unlike other factors involved in plant species distributions, it is global, permanently active and spatially continuous. Hence, the geographical variation of climate on predictable gradients such as altitude and latitude allows the analysis of the responses of species along ample ranges of environmental variation. Specifically, the study of plant populations' dynamics along climatic gradients has a great interest to predict likely responses to climate warming. Some studies have already raised the alarm about the upwards and polewards shifts of plant species ranges during the twentieth century as a consequence of the global warming [3]–[7]. By contrast, other authors have shown demographic compensations or species benefiting from increases in temperature or aridity [8]–[11].

The research of causal links between climate and plant population responses is particularly interesting for analysing the dynamics of marginal populations [12]. Species populations at the rear edge of their distribution area are considered an important source of natural history information under past environments [13], essential to estimate their evolutionary potential [14] and to establish proper management guidelines under current uncertainties. Preservation of these populations necessarily depends on the success of the current recruitment under the ongoing changing conditions. Juveniles of woody species are more susceptible to climate, and more specifically to extreme events [15], and respond quicker than adults to environmental changes [16]. Therefore, shifts in the recruitment pattern may reflect subtle differences in climate and their potential future responses. For instance, several authors have already reported increments in elevation for recruitment rates of some tree and shrub species compared to the mean range of the conspecific adult individuals [9], [17]–[19], combined with a replacement of species at lower areas [16], [18], [20]. This seems a response to a direct effect of the changing climate on recruitment over seedling and sapling performance (with higher stress caused by heat and/or drought), and indirect over the performance and mortality of the adult trees, the potential parents [17], [20]–[22]. Some of these effects have been evaluated on rear edge populations, but unfortunately on isolated populations. Furthermore, most of these works did not take into consideration the extreme complexity and heterogeneity of what are called marginal populations [18], [20]–[21].

In the Iberian Peninsula, Scots pine (*Pinus sylvestris* L.) is a clear example of a boreal species at the rear edge of a large distribution area, and, obviously, this pine has to face ecological conditions different from those at the centre of its distribution range. Studies from central and northern Europe pointed out low temperature as the main abiotic limiting factor for the recruitment [23]. Meanwhile, in Mediterranean areas the summer drought is the factor that mainly limits seedling survival and growth [24]. However, Iberian Scots pine populations face very different climatic conditions within this region. In addition to the expected latitudinal trend across the different mountain ranges where the species occurs in this rear edge (i.e. from Pyrenees to Sierra Nevada at the southernmost end of its distribution), there are finer scale gradients nested within each mountain range, which reproduce rear, optimum and leading edge conditions making the interpretation of what is currently occurring in the so-called rear edge extremely complex. At these finer scales, non-climate factors (both biotic and abiotic) can be at least as much relevant for species distribution as climate [25] by modulating the direct effect of regional climate on individuals [2].

Taking into account the predictions in Mediterranean areas in terms of increasing aridity and temperature [26], the population dynamics and mainly the recruitment of this pine may be even more strongly conditioned by the spatial heterogeneity at the local scale [12]. Several works have focused on the study of the spatial heterogeneity at microhabitat scale in order to disentangle the main factors influencing emergence and/or survival in Mediterranean areas for species at their rear edge [20], [27]–[31]. Most of these studies highlighted the decisive role that plant-plant interactions had over the recruitment patterns, specially the nurse role of shrubs ameliorating the harsh climatic conditions imposed by summer drought [24], [32]. Indeed, they all supported the non-random effect that local environmental conditions (biotic and abiotic factors) exert over the juveniles' survival and growth, leading to aggregated patterns in *safe sites* [31]. Particularly, studies addressing the recruitment of *P. sylvestris* in its southernmost distribution area have only considered these local factors neglecting the variability across the landscape [20], [24], [29], [33] (but see [22]). Thus, a critical question remaining is to explore whether juveniles of *P. sylvestris* respond to local scale conditions similarly across climatic and nested altitudinal gradients in different rear edge regions.

In the present study, we aimed to find out whether there are generalizable patterns at different spatial scales in the recruitment of Scots pine populations in its rear edge. Specifically, we explored the relative effect that climate and local factors may have over recruitment across several mountain ranges, surveying the complete altitudinal range of this pine species within them. The local variables comprised both biotic (cover of herbs, shrubs and adult individuals) and abiotic variables (slope, rockiness, light availability), describing not only conditions of the habitat (i.e. at the stand structural level), but also microhabitat conditions (i.e. at microsite scale). Moreover, we tested the effects of climate on local interactions, including plant-plant interactions (among juveniles and the pre-existing vegetation) to figure out whether climate exerts a multi-path impact over recruitment, not only directly over demography, but also indirectly interacting with local factors (at community level).

## Materials and Methods

### Study sites

**Figure 1:**
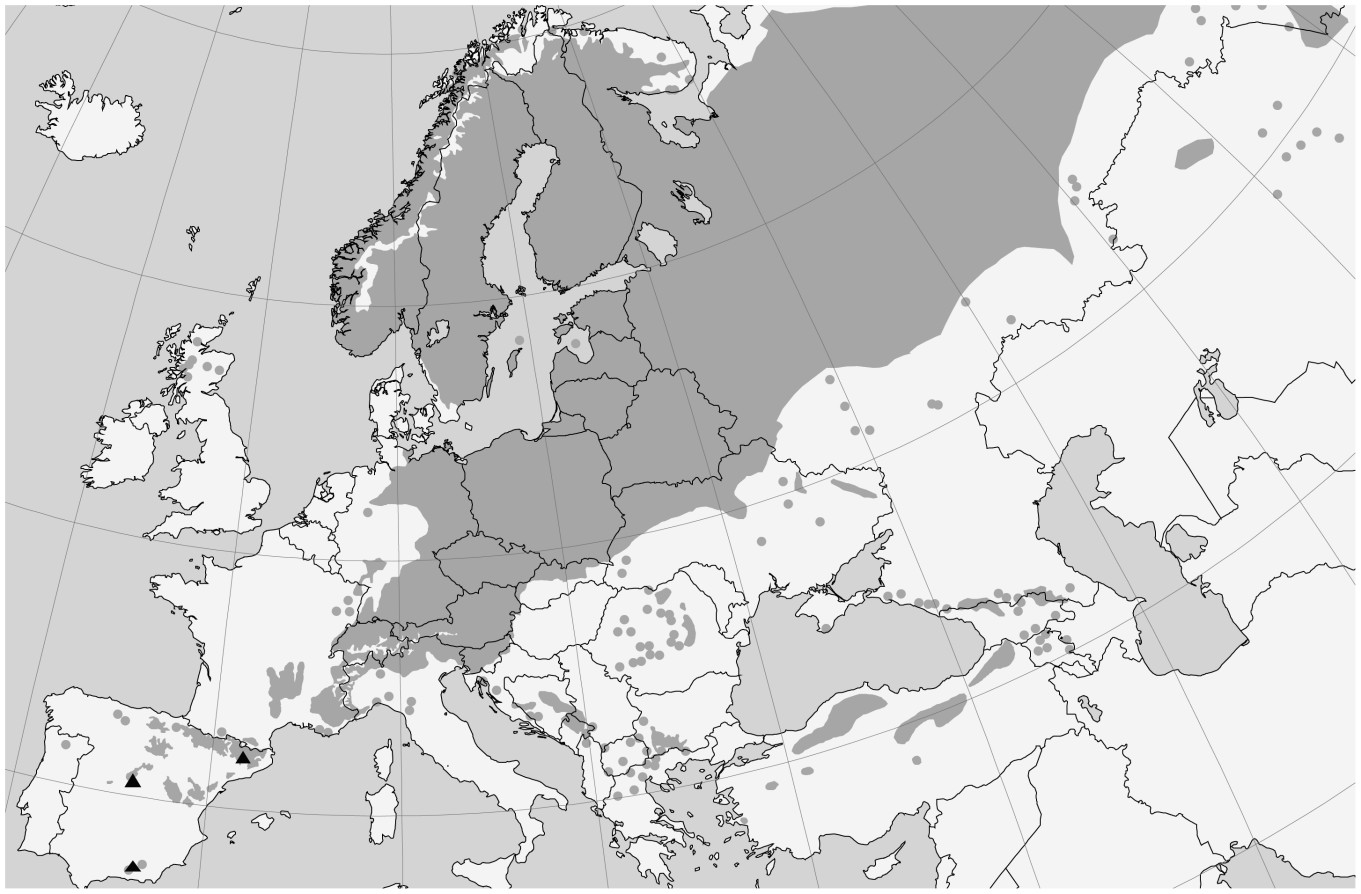
Location of the sampling sites (black triangles). Dark grey denotes distribution range of Scots pine (base map: www.euforgen.org).

The field work was conducted in three forests across a latitudinal gradient in Spain (Fig. 1). The northernmost stand was located in Arcalís, in Central Pyrenees (42° 22′ N, 1° 06′ E), with an altitudinal range from 670 to 1600 m. The central location was placed in Valsaín, on the north-facing slopes of Sierra de Guadarrama (40° 49′ N, 4° 01′ W), between 1130 to 1900 m. Both forests are pure Scots pine stands, with scattered individuals of other species mostly at lower altitudes, mainly *Quercus ilex* and *Q. pyrenaica* at Valsaín, and *Q. ilex*, *Q. humilis* and some planted *Pinus nigra* at Arcalís. These forests have had a long history of harvest and management. In Valsaín, the forest is managed under a group shelterwood system since long ago, always relying on natural regeneration, but no harvest has been accomplished in the sampled areas for the last 15 years. In the forest in Arcalís, logging activities took place in the past but have not been practiced since the last 30 years. In fact, current pine population exhibits natural regeneration and an uneven age and size structure.

The third location is Barranco del Espinar at Sierra Nevada National Park, the southernmost distribution area for Scots pine throughout its overall range (37° 06′ N, 3° 27′ W). The native *P. sylvestris* individuals are present from 1400 to 2100 m and coexist with other tree species, such as *Taxus baccata*, *Acer opalus* ssp. *granatense* or *Quercus ilex* at low tree density (around 114 trees ha^−1^). The area was traditionally grazed by livestock (goats and cattle), and currently the area is only grazed by increasing populations of wild ungulates, namely Spanish ibex (*Capra pyrenaica*). No specific permits were required for the described field studies, as the locations were not privately owned and there was no involvement of endangered or protected species. The authorities responsible of these areas were informed and they expressed their consent to this sampling.

### Sampling and data

We conducted the field work in late spring-early summer 2010 and 2011. We established six (in Valsaín) and five (in the other two sites) transects at different elevational levels, covering the whole altitudinal range of *P. sylvestris* in each site. The sampling design consisted of six plots at each altitudinal level, separated at least 100 m. Three plots were haphazardly located under fully canopied stands, and the other three in more open areas to include contrasting conditions within each forest and altitude, avoiding ravines, boulders or other geomorphologic elements which could bias our measurements. In the centre of every plot we established a 4×4 m frame and counted the number of seedlings recently emerged (from current year) and individuals older than a year and till 1.5 m high (hereafter saplings) in every 1 m^2^ subplot (16 within a plot). We estimated their age by counting the terminal bud scars (internodes) along the main stem, included the buried part, and we sorted them into four different age classes: 0) for seedlings; 1) for individuals between 1 and 2 years; 2) for 3 to 5 year-old individuals; and 3) for those over 5 years. The age of the individuals included in the last category was not fully dated due to the difficulty to distinguish scars in the field as individuals grow and become woodier, but it was verified that they exceeded 5 years. We also measured the shoot growth of saplings from previous years (2007, 2008 and 2009). Whenever juvenile density was under five individuals per plot, we enlarged the census area to a final plot of 6×6 m. In summary, we assessed the recruitment analysing three processes: emergence (inferred by seedlings abundance), seedling survival (inferred by the transition from seedlings to saplings) and primary growth of the saplings [34]. In early summer, the abundance of seedlings would mean *sensu stricto* the early survival of seedlings [34], but in the present study it has been considered a proxy of emergence as juveniles had not still coped with their main bottleneck: the summer drought.

The recorded variables represented biotic and abiotic environmental conditions assessed at different scales. At broad scale, we translated the variation in elevation and latitude into climatic variables using the Climatic Digital Atlas of the Iberian Peninsula [35].

At plot level (i.e. at habitat scale), we measured the slope and the light availability (GSF, global site factor), obtained by a hemispherical photograph taken at the centre of each plot [36]. In terms of biotic conditions, all adult trees (individuals of any species with diameter at breast height -dbh- over 7.5 cm) within a 10 m radius around the regeneration plot were mapped. Afterwards we calculated the basal area for every plot as an index of competition with adult trees. This radius size was consistent with that used in other tree competition studies [37]–[38].

At subplot level (microhabitat scale), we visually estimated the percentage of rocks, shrubs and herbs in every 1 m^2^. Finally, we assessed a potential fecundity index for each subplot considering the conspecific adult trees previously mapped, together with those bigger than 20 cm of dbh located between 10 m and 20 m from the plot centre. This index had two components (see [38], [39]): one related to the number of seeds that an adult tree can produce (seed rain), which is proportional to the tree size (dbh); and other that accounted for the seed dispersal capacity, which was assumed to decline exponentially as the distance to a specific tree increases.

### Statistical analyses and zero-inflated models (ZIP)

To evaluate the effects of climate on demography and performance of juveniles of *P. sylvestris* we carried out mixed models. The density of individuals, both seedlings and pooled saplings (age class was neglected due to the low figures in some categories), and the mean annual shoot growth were our response variables; and variables describing the environment (biotic and abiotic) at different scales were the explanatory ones (see list in Table S1). The interactions between temperature and the biotic factors (cover of co-occurring vegetation) and the quadratic term of temperature was included to consider non-monotonic responses along the gradient.

The density of seedlings and saplings per subplot (per m^2^) followed a Zero-Inflated Poisson distribution (ZIP), which accounts for an excess of zeros avoiding the underestimation of the number of zeros and the overestimation of large count occurrence [40], [41]. This ZIP consists of a binomial distribution model representing the occurrence (observing or not recruits), and a Poisson distribution model, conditional on the first, representing the abundance (number of recruits), both processes evaluated at the same time. A complete review of ZIP models for count data can be found in literature (e.g. [42], [43]). To fit models with ZIP distributions we used the SAS procedure NLMIXED (SAS Institute Inc. 2008. SAS/STAT 9.2) that allows the optimisation of our customized likelihood function.

We added random components into the intercepts of the linear predictors of the models [41] due to the hierarchical structure of the data, with likely different level of correlation among observations from the same site, transect or plot (nested design). The statistical procedure used in this study recommends the use of just one random effect due to the complexity that the likelihood function reaches and problems detected with the convergence of the model. Thus, we assumed site as a fixed effect, considering that this variable only counted with three levels. Regarding transect and plot, we preliminary fit the model including both effects separately, and we chose the model which harboured more variability (i.e. plot) using the Akaikés Information Criterion (AIC). Concerning fixed effects, we selected the best model following the principle of parsimony to find the simplest model that was not significantly worse than any more complicated one in terms of AIC.

Previously, in order to avoid multi-collinearity problems we checked the correlation among variables (Table S1). This analysis showed high correlation among potential fecundity index, basal area and GSF. Since Gómez-Aparicio [44] showed that in Mediterranean areas recruitment was more correlated to the best patches for emergence and survival than to seed rain -site instead of seed limitation-, and since competition among adults and juveniles is expected to be mainly related to light availability, we kept GSF as a proxy of the overstorey structure and discarded the other two predictors for ZIP models.

For analysing mean primary growth, we considered the same random and fixed variables (coupled with age class as a fixed factor) than those used for the juveniles' density; and again the selection criteria followed the principle of parsimony using the AIC as a measure of model fit. This analysis was also implemented using the SAS procedure NLMIXED, considering a normal distribution of the data after a log-transformation.

## Results

### Site characteristics and regeneration distribution

In terms of climate, the three sites were quite representative of the distribution area of Scots pine in Spain (Figure S1), but for precipitation in SN (p<0.001). The altitudinal range of the three sites was quite different, but there was no significant difference in terms of temperature among them.

**Figure 2:**
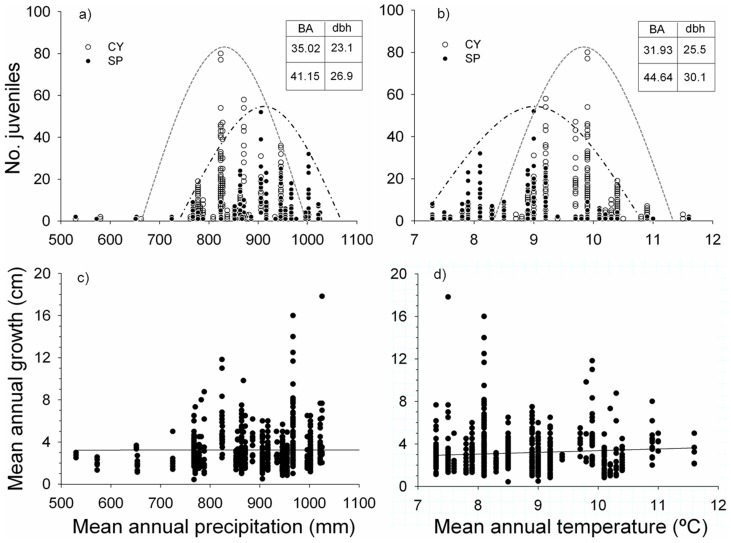
Distribution of current-year seedlings (CY) and juveniles older than a year (SP) according to a) the annual mean precipitation; and b) the mean annual temperature (with their envelope lines for each cohort). Distribution of mean growth according to c) the mean annual precipitation and d) mean annual temperature. (BA: mean basal area (m^2^ha^−1^); dbh: mean diameter at breast height (cm); both calculated within an interval around the optimum annual mean precipitation (±50 mm) and temperature (±0.5 °C), respectively).

**Figure 3:**
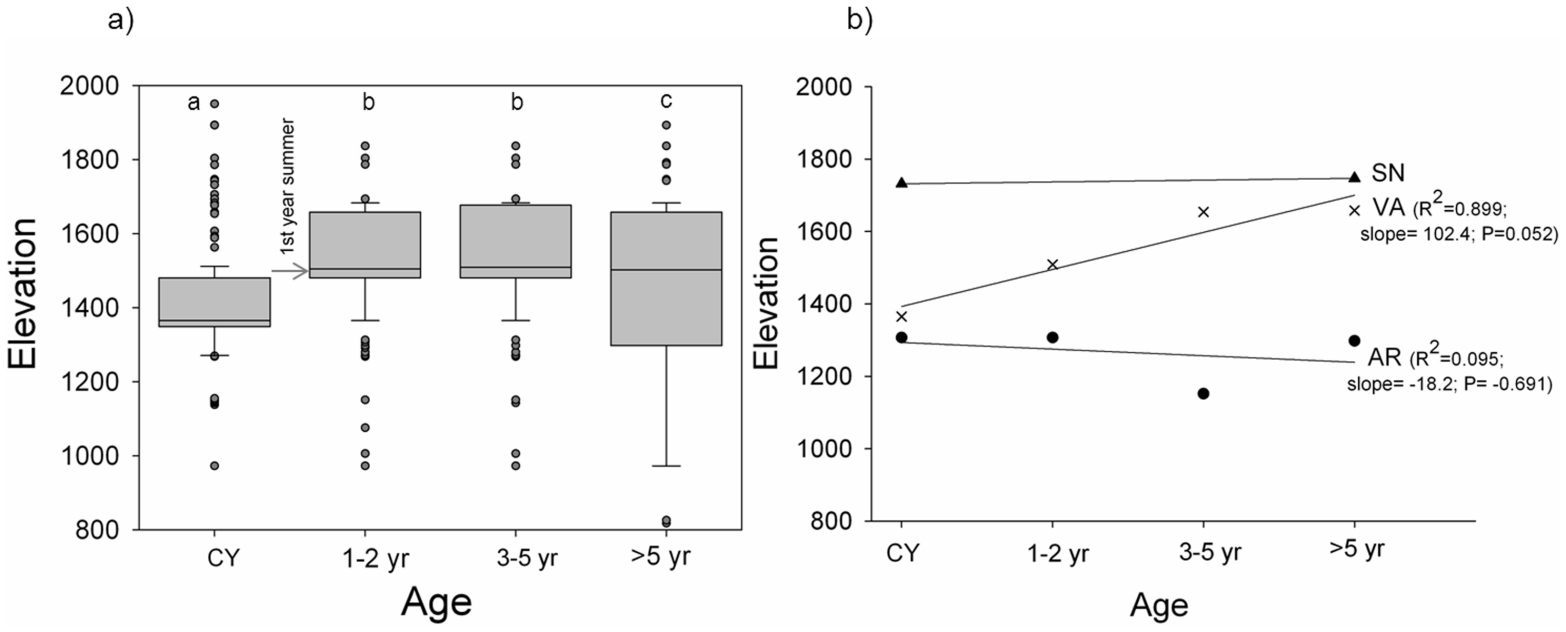
a) Altitudinal distribution of juveniles according to their age class and; b) altitudinal distribution of their medians according to each site. Different letters indicate significant differences (Kruskal-Wallis test)

The presence of juveniles was different among sites (p<0.001) (Table 1). Across the altitudinal gradients and sites, the distribution of juveniles varied non-linearly. The optimum climatic conditions for pooled seedlings appeared around 823 mm and 9.9°C; while for pooled saplings the presence was higher at lower temperatures and higher precipitation, i.e. around 905 mm and 9°C (Fig. 2). These climatic divergences were the result of the altitudinal gap between the optima of both cohorts (Fig. 3), showing a higher frequency of seedlings and saplings at 1362 m and 1490 m respectively (optimum altitude weighted by site).

**Table 1:** Descriptive variables measured in the sampling plots in the three study sites.

	Valsaín	Arcalís	Sierra Nevada
Latitude	40°49′ N	42°22′N	37°06′N
Longitude	4°01′W	1°06′E	3°27′W
Altitudinal range (m)	1138–1837	672–1597	1425–1989
T range (°C)	7.3–10.9	7.3–11.6	8.7–11.5
Mean T (°C)	9.3 (1.3)	9.6 (1.6)	9.9 (1.0)
P range (mm)	762.7–1025.4	572.3–955.1	529.7–867.0
Mean P (mm)	873.6 (91.0)	827.2 (109.7)	691.4 (102.7)
Mean slope (%)	15.7(10.3)	45.1 (19.8)	35.0 (12.9)
Mean cover shrubs (%)	7.5 (15.8)	25.7 (26.2)	26.7 (28.1)
Mean cover herbs (%)	39.0 (32.7)	28.2 (26.5)	20.3 (21.5)
Mean cover rocks (%)	2.5 (7.2)	9.7 (15.3)	14.0 (20.0)
Mean tree density (pines ha^−1^)	298.9 (255.4)	603.7 (473.6)	165.5 (117.1)
Mean basal area (m^2^ ha^−1^)	36.8 (22.7)	31.8 (13.8)	19.7 (14.4)
Mean dbh of adult pines (cm)	36.7 (11.8)	22.9(5.7)	37.8 (27.3)
Mean GSF	0.39(0.24)	0.27 (0.12)	0.43 (0.28)
No. observed CY	3794	77	45
No. observed SP	1211	197	7
No. plots with zero recruits	1	11	19

Standard deviations are shown in brackets.

T: mean annual temperature, P: annual mean precipitation; dbh: diameter at breast height; GSF: global site factor, CY: current-year seedlings, SP: juveniles older than a year.

### Factors affecting recruitment

Table 2 shows the variables included in the final ZIP models. It is remarkable that these models showed the significant variables affecting the occurrence (in particular the “no event”, i.e. the absence) and abundance of juveniles independently.

**Table 2:** Zero-Inflated Poisson Mixed Models for the occurrence (absence)* and abundance of recently emerged seedlings and juveniles older than a year (saplings).

		Occurrence Model*				Abundance Model			
	Variables	Estimate	SE	t value	Pr>|t|	Estimate	SE	t value	Pr>|t|
Seedlings	Constant	78.5909	32.5935	2.41	0.0179	−84.6586	11.1953	−7.56	<.0001
*(AIC = −12038)*	Site 2	−4.1053	1.4245	−2.88	0.0049	4.5493	0.4974	9.15	<.0001
*(EF = 0.742)*	Site 3	1.5625	1.41	1.11	0.2707	2.6094	0.5332	4.89	<.0001
*(ΔAIC = 125)*	T	−15.7198	6.8578	−2.29	0.0242	17.3094	2.3224	7.45	<.0001
	T^2^	0.7892	0.3609	2.19	0.0313	−0.9152	0.1217	−7.52	<.0001
	P	-	-	-	-	-	-	-	-
	Slope	-	-	-	-	0.03153	0.01165	2.71	0.0081
	Herbs	-	-	-	-	−0.03711	0.01675	−2.22	0.0292
	Shrubs	-	-	-	-	-	-	-	-
	Rocks	-	-	-	-	-	-	-	-
	GSF	-	-	-	-	-	-	-	-
	Herbs xT	-	-	-	-	0.004191	0.00173	2.42	0.0174
	Shrubs x T	0.002273	0.001005	2.26	0.0261	-	-	-	-
	GSF x T	-	-	-	-	-	-	-	-
Saplings	Constant	5.2785	1.2245	4.31	<.0001	−32.0442	10.2046	−3.14	0.0023
*(AIC = −1646)*	Site 2	−5.1948	1.1335	−4.58	<.0001	1.2807	0.949	1.35	0.1805
*(EF = 0.598)*	Site 3	−4.6633	1.1484	−4.06	0.0001	1.229	0.9268	1.33	0.1882
*(ΔAIC = 87)*	T	-	-	-	-	7.0072	2.1234	3.3	0.0014
	T^2^	-	-	-	-	−0.4299	0.1147	−3.75	0.0003
	P	-	-	-	-	0.00431	0.001508	2.86	0.0053
	Slope	−0.03114	0.02128	−1.46	0.1468	−0.01973	0.01113	−1.77	0.0796
	Herbs	0.01801	0.005788	3.11	0.0025	−0.0734	0.0203	−3.62	0.0005
	Shrubs	-	-	-	-	-	-	-	-
	Rocks	-	-	-	-	-	-	-	-
	GSF	-	-	-	-	-	-	-	-
	Herbs xT	-	-	-	-	0.007874	0.002344	3.36	0.0011
	Shrubs x T	-	-	-	-	-	-	-	-
	GSF x T	-	-	-	-	-	-	-	-

Every variable introduced in the models is listed, but only the estimates for the variables included in the final model are shown.

Site 1: Valsaín, Site 2: Arcalís, Site 3: Sierra Nevada. T: mean annual temperature, P: annual mean precipitation, GSF: Global site factor; EF: modelling efficiency; ΔAIC: difference in AIC referred to saturated model.

* The parameters estimated in the binary part are referred to the probability *P* (finding no recruit) assessment, being 1-*P* the probability of presence.

**Figure 4:**
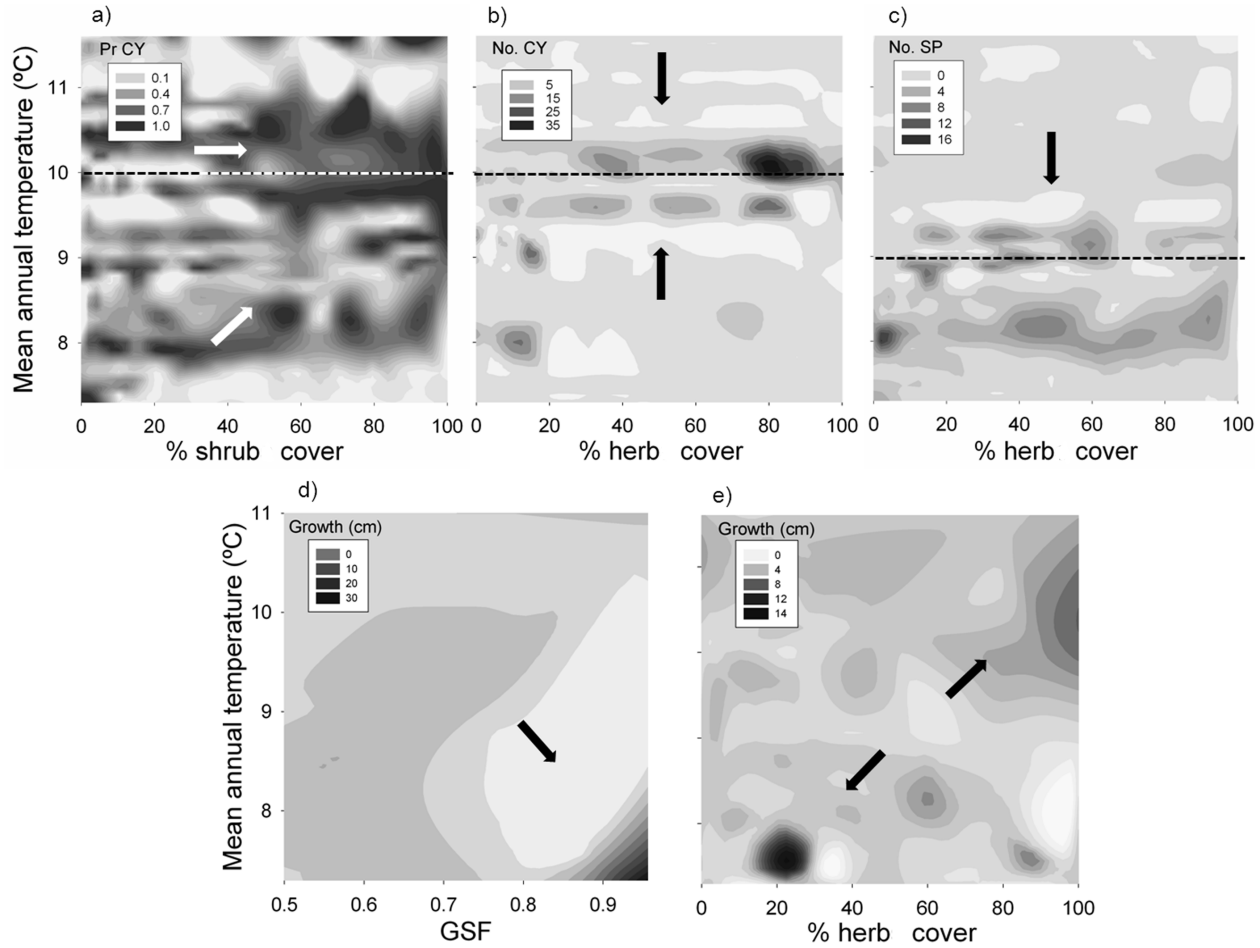
Significant interactions among mean annual temperature and: a) cover of shrubs affecting the probability of current-year seedlings' presence (Pr CY); b) herb cover affecting the abundance of seedlings (No. CY); c) herbs over the abundance of saplings (No. SP); d) GSF affecting mean growth of SP; and e) herbs on mean growth of saplings. Dotted line shows the mean annual temperature at which the number of CY (a, b) and SP (c) were more frequent, respectively.

The selected model for seedlings showed a clear quadratic effect of mean annual temperature with significant values for both parts of the model (occurrence and abundance). The difference among sites (site factor) in terms of presence and abundance was also significant. The slope affected positively, and the percentage of herbaceous plants negatively to the density of seedlings (abundance model). We observed that temperature also interacted with other variables. Thus, the interaction between temperature and the cover of shrubs was significant (occurrence model), with an increasing probability of observing seedlings in places with a high cover of shrubs as annual temperature increased (Fig. 4a). Temperature also interacted with the cover of herbs, increasing its negative effect at the extreme low and high temperatures (abundance model) (Fig. 4b).

The final model for saplings also included the site as a significant factor (occurrence model) and the climatic variables (abundance model): annual temperature again showed a quadratic significant effect and annual precipitation had a positive effect. The cover occupied by herbs affected negatively in both parts of the model, and interacted with temperature being less important at lower temperatures (Fig. 4c).

### Factors affecting sapling growth

Mean values of growth seemed to stay approximately constant with temperature and precipitation (Table 3; Figs. 2c, 2d), but it can be observed that the number of juveniles and the dispersion of the data was lower under more stressful conditions (higher temperatures and lower precipitation) (Figs. 2c, 2d). High light availability (GSF), rockiness and a low herb cover led to significant greater shoot elongations (Table 3). In addition, GSF and herbs interacted with annual temperature. The positive effect of light on growth was higher at low temperatures (Fig. 4d); whereas growth showed a more complex response when temperature interacted with herbs: at low temperatures juveniles grew more with low percentage of herbaceous, but also at high temperatures with high cover of herbs (Fig. 4e). Finally, the age of the saplings was also significant, showing higher mean growth for juveniles over 5 years.

**Table 3:** No Linear Mixed Model for the mean shoot growth of juveniles.

Variable	Estimate	SE	t value	Pr>|t|	AIC	ΔAIC	EF
Constant	2.1146	0.4303	4391	<.0001	3717.2	209.6	0.407
Age class 1	−0.5550	0.1477	−3.76	0.0005			
Age class 2	−1.4442	0.1556	−9.28	<.0001			
T	-	-	-	-			
T^2^	-	-	-	-			
P	-	-	-	-			
Slope	-	-	-	-			
Herbs	−0.09288	0.02941	−3.16	0.0029			
Shrubs	-	-	-	-			
Rocks	0.01521-	0.006872	2.21	0.0321			
GSF	16.0222	4.2561	3.76	0.0005			
Herbs x T	0.01051	3.11	0.0033	0.0033			
Shrubs x T	-	-	-	-			
GSF x T	−1.2034	0.4558	−2.64	<.0001			

Every variable introduced in the models is listed, but only the estimates for the variables included in the final model are shown.

T: mean annual temperature; P: annual mean precipitation; GSF: global site factor; Age class 1: 1–2 years old; Age class 2: 3–5 years old; Age class 3: >5 years old; ΔAIC: AIC increment referred to saturated model; EF: modelling efficiency.

## Discussion

### Effects of climate on demography

In the present study we have accomplished, for the first time, a nested survey of the recruitment pattern of Scots pine along altitudinal gradients at three different mountain ranges throughout the southernmost limit of its distribution area. Despite the different altitudinal range and characteristics of each site, the altitudinal gradient within each mountain range reproduced the rear, optimum and leading edges of the study populations under similar temperature ranges which led to a similar distribution pattern of juveniles in the different rear edge regions. Thus, the results showed a clear non-linear effect of temperature on the distributions of both cohorts, with an optimum temperature range where juveniles were more frequent, above and below which the number dropped gradually similarly to results in previous studies [9], [17], [19]. These higher frequencies of juveniles tallied with altitudinal levels that were above the mean elevation of the overall range of the adult stands (1330 m), though the differences among seedlings and saplings were remarkable. Regarding the former, the optimum of their distribution is close to the mean altitudinal range of adults. Meanwhile, the optimum for saplings' density was over 100 m skewed towards higher altitudes (lower temperatures and higher precipitation) compared to seedlings. In fact, considering the age classes of juveniles, there was an abrupt transition towards higher altitudes after the first year. This transition from seedlings to sapling is acknowledged as the main bottleneck for Scots pine recruitment with reported mortality rates around 80–100% after the first summer drought [15], [27], [31]. Thus, our data suggest that first summer survival may principally occur at locations with higher precipitation and lower temperature than those where more seedlings emerged. Likewise, other studies have already highlighted the positive role of water availability (related with precipitation and temperature) for the survival of Scots pine seedlings in Mediterranean areas [27], [31].

### Effect of climate on plant-plant relationships

The surrounding structure of adult trees have a great relevance on regeneration patterns, in terms of seed source and mitigation of heat stress conditions (positive effects); or in terms of competition for light (negative effect) [27], [33], [39]. Thus, the direction of the net effect of overstorey structure on recruitment will depend on species-specific trade-offs or on the specific limiting factor within a system. Previous studies pointed out the light availability (as a direct consequence of the overstorey structure) as one of the best predictors for recruit distribution [29], [45], [46]. In our study case, we found no significant net effect of light availability on the abundance of seedlings or saplings. This might be due to the negligible relative effect of seed rain distribution in pure stands, or the heat stress amelioration provided by adult trees partially offsetting the lack of light. Nevertheless, our results did reveal the critical positive effect that light availability had over the shoot growth of saplings, as an indicator of their current performance and potential success within the stand [30], [45]. This effect was more marked at sites with lower temperatures, where the stress caused by heat and drought dropped.

Many works have investigated plant-plant interactions along gradients of environmental severity [47]. In Mediterranean areas, compelling evidence about the nurse effect exerted by shrubs on recruits has already been reported by different studies [27], [32], [46], [48]. Likewise, our results showed a significant effect of climate (temperature) on the interaction between seedlings and shrubs: the probability of finding them was higher near shrubs as mean temperature increased, supporting the idea of stress amelioration underneath. Surprisingly, we did not detect any effect of the shrub presence in relation to saplings, as previous research has reported in Sierra Nevada [24], [27]. This lack of significance may suggest either that the benefits provided by the nurse plant may not overcome its own resource uptake [49]; or may be due to different lifespan of shrubs and tree juveniles that may hide interactions in the past. Additionally, the specific shrub species in each site and their peculiarities, not considered in the present study (e.g. allelopathic effects, architecture, herbivory appeal) may play a crucial role in fading or neglecting this nursing effect [32], [50].

The herbaceous layer constitutes a physical barrier preventing seeds from contacting the mineral soil [24], and competes with seedlings for resources [51]. This is in accordance with the negative effect that herb cover had on seedling emergence and survival in our study. This effect varied with temperature, shrinking at less stressful sites, i.e. at intermediate temperatures for emergence (seedlings); and at lower temperatures for survival (saplings), precisely where precipitation was higher. This might indicate that the competition is harder under harsher conditions, subscribing evidence that negative relationships do not always predominate under less stressful conditions [52]. Cover of herbs significantly hampered the mean growth of saplings, likely due to competition for water as well [51], [33], being less evident in places with cooler temperatures (less stressful conditions). However, at warmer sites, growth increased with a higher cover of herbs, suggesting a stimulation of shoot elongation in more stressful sites, in order to escape from competition in the same way as shade conditions do [53], [54].

### Complex scenarios for Scots pine: considerations for management

Recruitment of Scots pine in the Iberian Peninsula is episodic, especially dependant on the climatic conditions during critical stages of seedling establishment or the availability of *safe sites*. In Mediterranean areas, rare wet summers entail the opportunity for high sporadic establishment [55]; whereas episodic events of severe drought occasionally cause massive seedling mortality [56]. Since these extreme events are predicted to become more frequent [26], it seems crucial to work on the preservation or promotion of these *safe sites*. The results of the present study showed that seedlings' emergence in southern populations of *P. sylvestris* booms at warmer temperatures than survival, and thus subsequent seedlings' success would rely on higher water availability and less heat-stress. Based on our results and in accordance with other studies, the partial removal of the herbaceous vegetation may ameliorate the competition for the scarce water [33], [57] and consequently boost seedling survival and growth. Moreover, the promotion of shrubs as nurse plants, not only may mitigate the summer stress for seedlings [24], [48], but also protect juveniles against mechanic damages such as grazing [50].

Nevertheless, management in Mediterranean areas should not only be based on silvicultural manuals. Silvicultural strategies must be tailor-made according to the idiosyncrasy of each place. Our results showed differences in the recruitment pattern among the three sites, probably due to multiple and diverse reasons. For instance, Sierra Nevada represents the most stressful site in climatic terms, with low density of adult trees and an important potential damage to juveniles by wild animals' browsing [58]. Therefore, operations aimed at the avoidance of the senescence of the existing adult trees and the promotion of the protective shrub layer may help to preserve stands with similar characteristics. The northernmost location, Arcalís, harbours other peculiarities. Galiano, Martínez-Vilalta & Lloret [20] suggested that management abandonment in the last decades (with consequent high tree density), might have favoured a poorer performance of adult trees under extreme events, constraining partially the recruitment in this area. Thus, a sustainable thinning may ameliorate conspecific competition for water and improve the performance of potential parents [59]. Finally, in the third stand, Valsaín, recruitment does not seem to be compromised. This may surely be linked to the higher precipitation records, but also to the regular management that so far has guaranteed the presence of mature and vigorous trees as seed source and provided appropriate size gaps for seedling establishment.

## Conclusion

The consideration of environmental variables at different scales constitutes a step forward to tackle the heterogeneity and captures the potential variability of recruitment across the landscape. Particularly interesting is to consider different elevation gradients (three sites in this study) within the overall rear edge area of any species, because they in turn reproduced the leading and rear edge conditions. Thus, our results have shown the general incidence of climate directly and indirectly on recruitment from demographic, performance and community standpoints, despite the idiosyncrasy of each site. This pattern may be useful for disentangling the potential effect of climate change on Scots pine recruitment and for establishing generalizable criteria for management in these marginal populations. Moreover, the consideration and assimilation of local biotic and abiotic interactions is essential to reach sustainable management for populations of *P. sylvestris*, but also to improve our understanding of the dynamics of these marginal populations. Therefore, we advocate further studies at individual and at community level, both in the field or in green houses, in order to understand the non-linear responses of communities to combined effects (climatic and non-climatic) and to deepen into their influence over the performance of the juveniles of these marginal populations.

## Supporting Information

Figure S1 Histograms of frequencies of the plots belonging to the III Spanish National Forest Inventory with *Pinus sylvestris* as a dominant tree, according to their mean annual temperature and precipitation [35]. The grey bars show where our sampling sites belong (AR: Arcalís, VA: Valsaín; SN: Sierra Nevada).
(DOCX)

Table S1 Spearman correlation coefficients among the variables measured in the study plots (T: mean annual temperature; P: annual mean precipitation; GSF: global site factor; FI: potential fecundity index; BA: basal area).
(DOCX)
