# Supplementary figures and images for: Direct and Indirect Effects of Climate on Demography and Early Growth of Pinus sylvestris at the Rear Edge: Changing Roles of Biotic and Abiotic Factors
Source: PLoS One. 2013 Mar 26;8(3):e59824. doi: 10.1371/journal.pone.0059824 (PMC3608533; doi:10.1371/journal.pone.0059824)

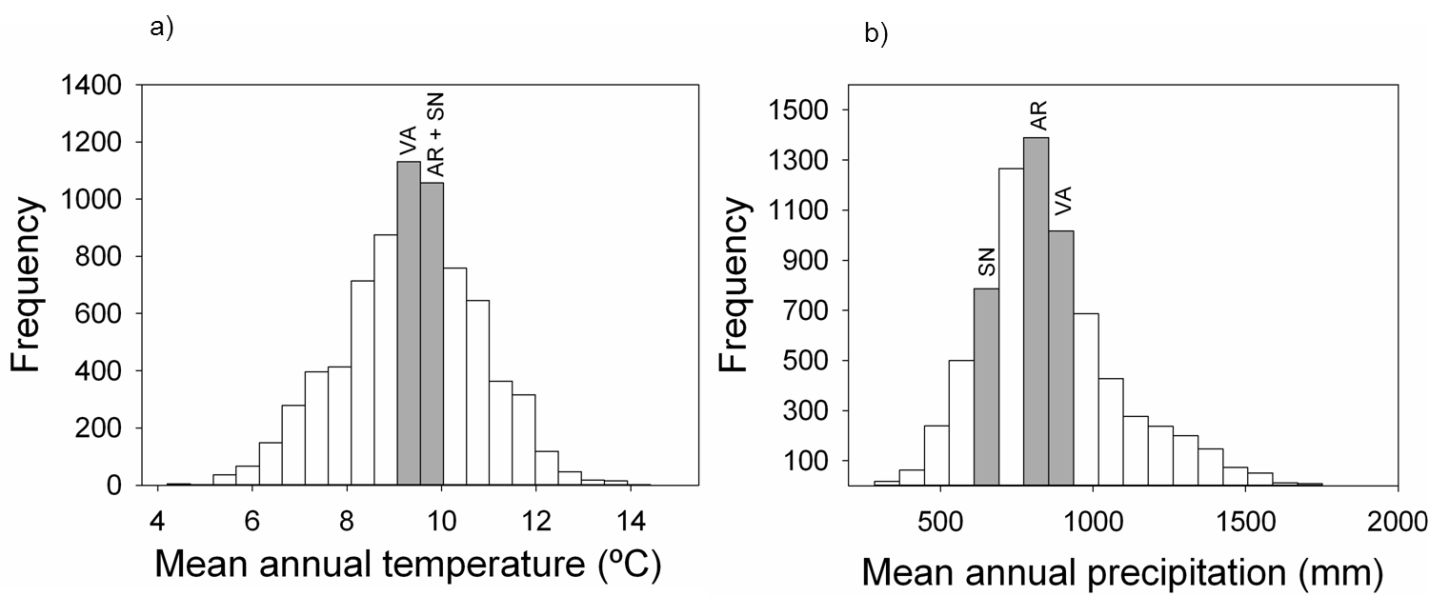


**Figure S1**.

Supplement: Figure S1 — Histograms of frequencies of the plots belonging to the III Spanish National Forest Inventory with Pinus sylvestris as a dominant tree, according to their mean annual temperature and precipitation [35]. The grey bars show where our sampling sites belong (AR: Arcalís, VA: Valsaín; SN: Sierra Nevada). (DOCX) [file pone.0059824.s001.docx]
