# Supplementary material for: Direct and Indirect Effects of Climate on Demography and Early Growth of Pinus sylvestris at the Rear Edge: Changing Roles of Biotic and Abiotic Factors
Source: PLoS One. 2013 Mar 26;8(3):e59824. doi: 10.1371/journal.pone.0059824 (PMC3608533; doi:10.1371/journal.pone.0059824)
Supplement: Table S1 — Spearman correlation coefficients among the variables measured in the study plots (T: mean annual temperature; P: annual mean precipitation; GSF: global site factor; FI: potential fecundity index; BA: basal area). (DOCX) [file pone.0059824.s002.docx]

**Table S1**.

|  | T | P | Slope | Shrubs | Herbs | Rocks | GSF | FI | BA |
| --- | --- | --- | --- | --- | --- | --- | --- | --- | --- |
| T | 1,000 | ,040 | ,085^**^ | ,341^**^ | -,322^**^ | ,155^**^ | ,137^**^ | -,215^**^ | -,405^**^ |
| P | - | 1,000 | -,031 | -,259^**^ | ,014 | -,101^**^ | ,004 | ,063^**^ | ,203^**^ |
| Slope | - | - | 1,000 | ,315^**^ | -,046 | ,255^**^ | -,042 | -,020 | -,118^**^ |
| Shrubs | - | - | - | 1,000 | -,172^**^ | ,230^**^ | ,147^**^ | -,214^**^ | -,349^**^ |
| Herbs | - | - | - | - | 1,000 | -,127^**^ | ,057^*^ | -,145^**^ | ,055^*^ |
| Rocks | - | - | - | - | - | 1,000 | ,235^**^ | -,287^**^ | -,420^**^ |
| GSF | - | - | - | - | - | - | 1,000 | **-,548^**^** | **-,539^**^** |
| FI | - | - | - | - | - | - | - | 1,000 | **,641^**^** |
| BA | - | - | - | - | - | - | - | - | 1,000 |
